# Crystal structure, Hirshfeld surface analysis and DFT study of *N*-(2-nitro­phen­yl)male­imide

**DOI:** 10.1107/S2056989024000926

**Published:** 2024-02-02

**Authors:** Maribel Montoya-Garcia, Héctor Cortes-Hernandez, Richard D’Vries, Hoover Valencia-Sanchez

**Affiliations:** a Grupo de Investigación en Fotocatálisis y Estado Sólido GIFES, Escuela de Química, Universidad Tecnológica de Pereira, Carrera 27 10-02, Pereira, Colombia; b Facultad de Ciencias Naturales, Exactas y de la Educación, Departamento de Química, Universidad del Cauca, Calle 5 4-70, Popayán, Colombia; University of Buenos Aires, Argentina

**Keywords:** crystal structure, nitro­phenyl­male­imide, Hirsfeld surface analysis, 1-(2-nitro­phen­yl)-1*H*-pyrrole-2,5-dione

## Abstract

The title compound [systematic name: 1-(2-nitro­phen­yl)pyrrole-2,5-dione], C_10_H_6_N_2_O_4_, crystallizes in the monoclinic system (space group *P*2_1_/*n*) with two mol­ecules in the asymmetric unit, which are linked by C—H⋯O hydrogen bonds. Hirshfeld surface analysis showed that the most significant contributions to the crystal packing are from H⋯O/O⋯H, H⋯C/C⋯H and H⋯H inter­actions, which contribute 54.7%, 15.2% and 15.6%, respectively. A DFT study was conducted using three different levels of theory [(B3LYP/6–311+G(*d,p*), wB97XD/Def2TZVPP and LC-wpbe/6–311(*2 d,2p*)] in order to determine the stability, structural and electronic properties of the title mol­ecule with a view to its potential applications and photochemical and copolymer properties.

## Abstract 2

The title compound crystallizes in the monoclinic system (space group *P*2_1_/*n*) with two mol­ecules in the asymmetric unit, which are linked by C—H⋯O hydrogen bonds. Hirshfeld surface analysis showed that the most significant contributions to the crystal packing are from H⋯O/O⋯H, H⋯C/C⋯H and H⋯H inter­actions. A DFT study was conducted using three different levels of theory.

## 1. Chemical context

1-(2-Nitro­phen­yl)pyrrole-2,5-dione is a compound derived from *N*-aryl male­imide (cyclic *N*-imides), with the –CO—N(*R*)—CO– functional group, where *R* is an aryl group (Hargreaves *et al.*, 1970 ▸) . *N*-phenyl­male­imides substituted by the N atom present various reactivity and photochemical properties that depend on the substituent group and the torsion angle between imide and benzene rings. These mol­ecules have been used as a copolymer, providing greater structural rigidity, increase in dielectric properties and thermal stability compared to the unreacted polymer (Mejia *et al.*, 2021 ▸; Shi *et al.*, 2020 ▸). The importance of these mol­ecules is due to the potential reactivity of the double bonds that act as dienophiles, promoting Diels–Alder reactions for the formation of new organic mol­ecules (Galkin *et al.*, 2022 ▸; Bastin *et al.*, 2019 ▸). Likewise, these families of compounds present good fungicidal properties against human pathogenic fungi, anti-leukemia activity, and differential cytotoxicity against cancer cells, among other biological activities (Paprocka *et al.* 2022 ▸; Mutlaq *et al.*, 2021 ▸; Ali *et al.*, 2017 ▸; Chen *et al.*, 2017 ▸). 
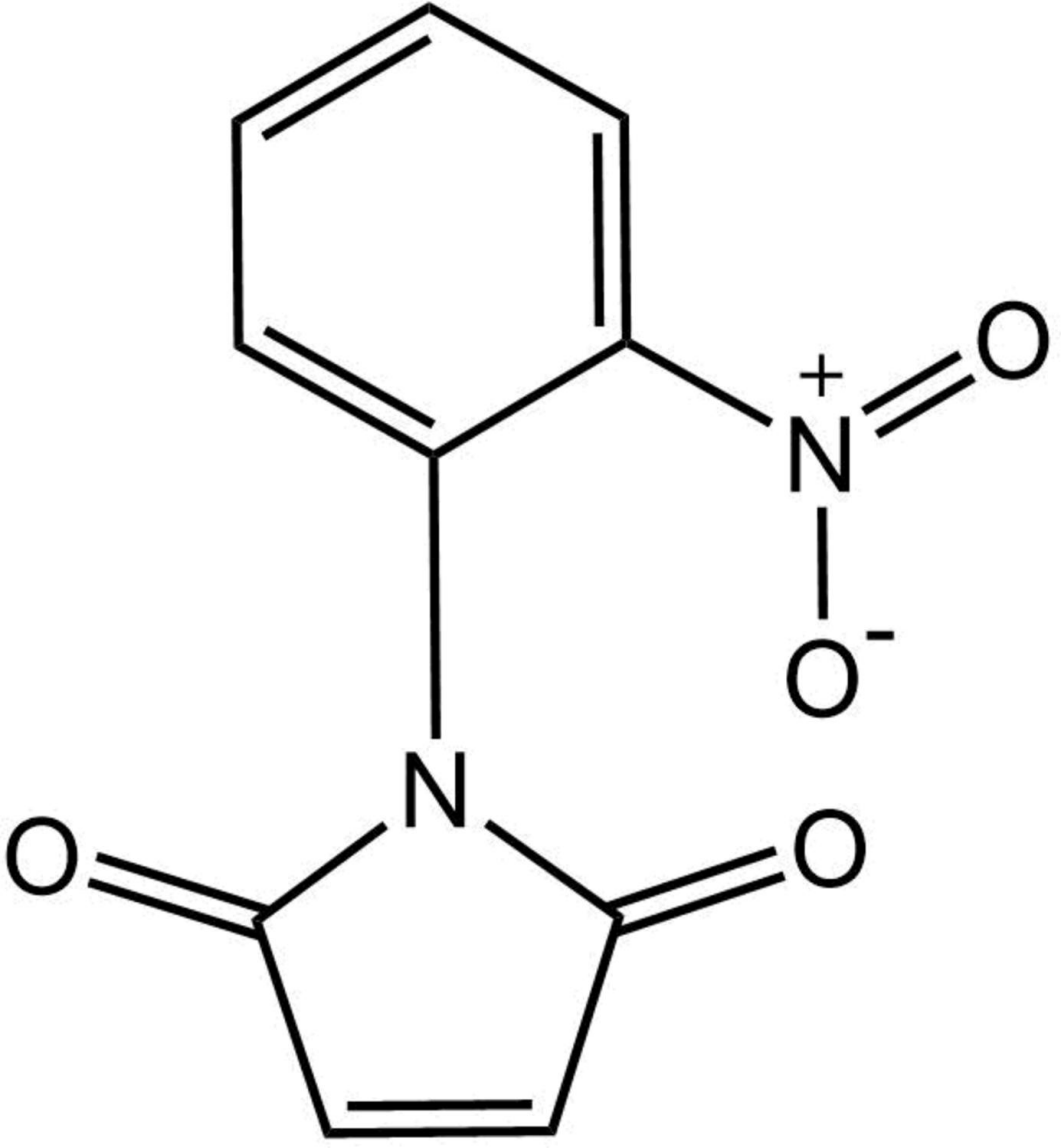


*N*-2-nitro­phenyl­male­imide [1-(2-nitro­phen­yl)pyrrole-2,5-dione], commonly called *N*-*ortho*-nitro­phenyl­male­imide, is used in homopolymers and copolymers with methyl methacrylate with excellent thermal stability, high polydispersity, and solubility in non-polar and moderately polar solvents. However, the substitution of the nitro group in the *ortho* position causes intra­molecular ring repulsion or steric hindrance with the male­imide ring (Kumar & Jagrati, 2023 ▸; Kumar 2022 ▸). Recently, in our group, a new water-friendly supra­molecular polymeric material obtained from the blend of isomers of nitro­phenyl­male­imide and carb­oxy-methyl­cellulose (CMC) was reported (García *et al.*, 2023 ▸). The polymers obtained present inter­esting properties such as high viscosity, resistance to acids, bases, and oxidant substances; also, this material presents an increase in thermal properties compared to CMC, and good biodegradability. However, among the polymers obtained, those synthesized from *ortho*-male­imides do not show good properties because of the repulsion of the nitro group with the imide ring, which affects the formation of hydrogen bonds (García *et al.*, 2023 ▸). Likewise, in a theoretical study of 43 mol­ecules of substituted *N*-phenyl­male­imides (including 2-nitro­phenyl­male­imide) in different positions using the B3LYP/ 6-311+G (*d*,*p*) method, it was found that the torsion angle affects the structural, electronic and energetic properties. Besides, the values of the global and local reactivity descriptors depend on the type of substituent (electron donor or acceptor groups). The substitution of the nitro group in the *ortho* position has greater global hardness and lower electrophilicity values than the *meta* and *para* isomers, suggesting a lower reactivity than for its isomers (Cortes & Castro, 2016 ▸). Continuing with the development of the synthetic methodology for obtaining all the isomers of nitro­phenyl­male­imide (Cortes & Castro, 2016 ▸; Moreno-Fuquen *et al.*, 2003 ▸, 2006 ▸), this work presents the synthesis, characterization by single-crystal X-ray diffraction, and analysis of supra­molecular inter­actions by Hirshfeld surface analysis from the structural data. In addition, theoretical calculations of structural and electronic properties were performed by density functional theory (DFT). Finally, the effect of the repulsion of the nitro group that affects the physical and chemical properties was examined.

## 2. Structural commentary

**Figure 1:**
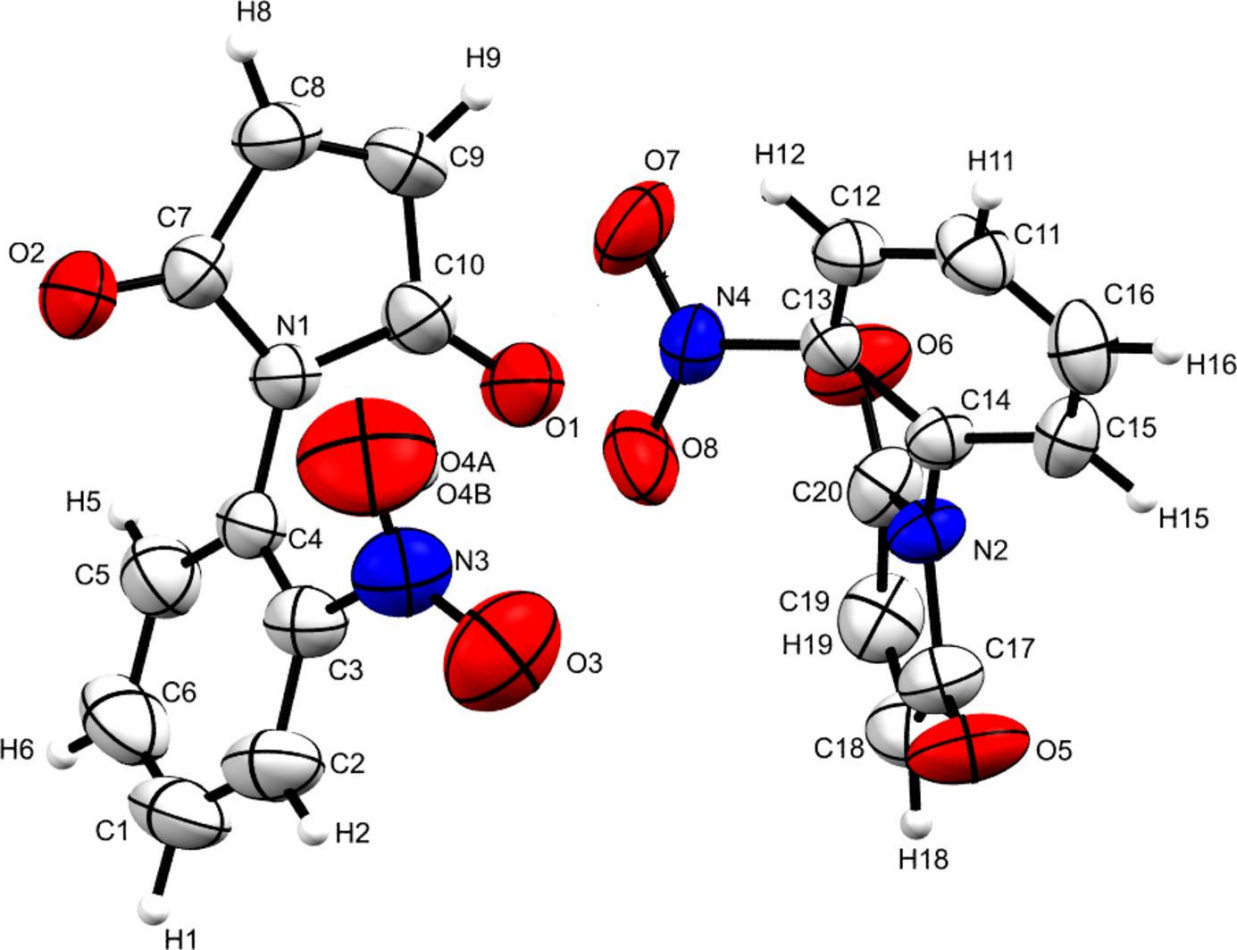
The mol­ecular structure of the title compound, with displacement ellipsoids drawn at the 50% probability level. Only one component of the disordered O4 atom is shown for clarity.

The asymmetric unit is formed by two independent mol­ecules (Fig. 1 ▸). Each mol­ecule consists of fused benzene and male­imide rings. In both conformers, a large dihedral angle is subtended between the rings with values of 73.94 (2)° for the C1–C6 and N1/C7–C10 rings and 55.02 (2)° for the C11–C16 and N2/C17–C20rings. A slight difference is observed in the torsion angle between the aromatic ring and the nitro group in the two conformers with values of 37.8 (3) and 38.8 (2)° for C2—C3—N3—O3 and C12—C13—N4—O8, respectively.

## 3. Supra­molecular features

**Figure 2:**
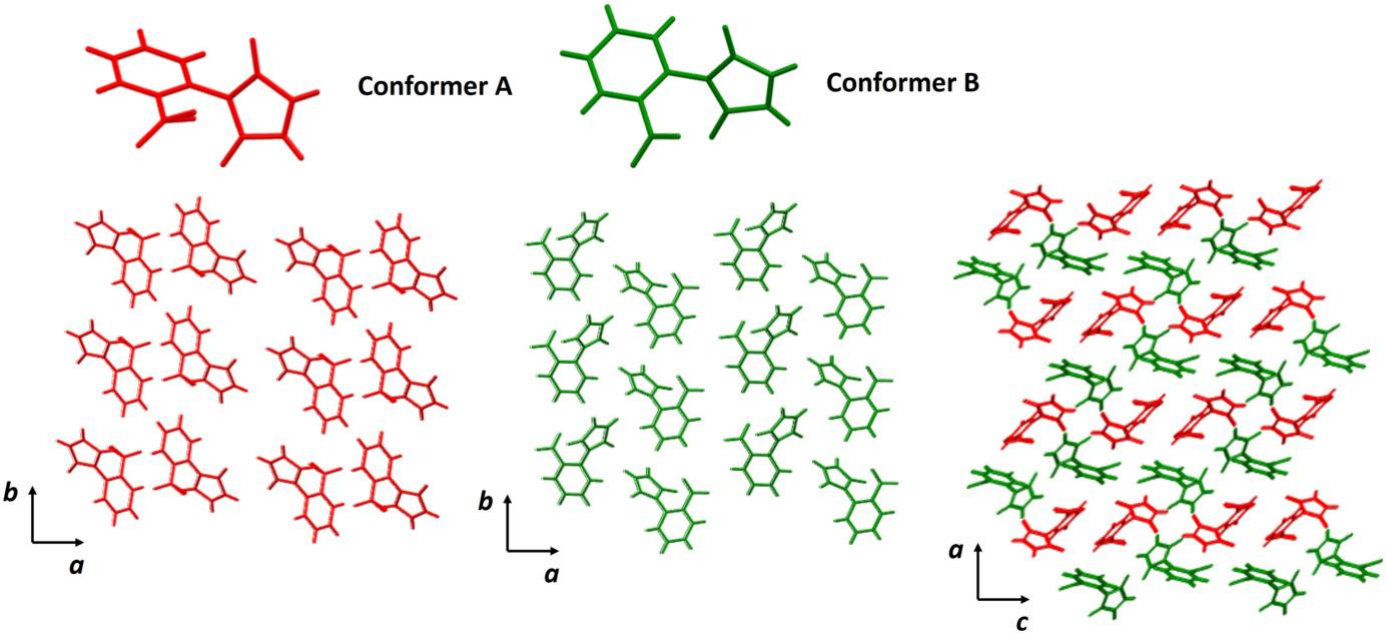
Conformers *A* and *B* of *o*-nitro­phenyl­male­imide.

In the crystal, the two conformers are arranged in lamellae in the (110) plane, with conformers *A* being linked along the *a-* and *b*-axis directions by C2—H2⋯O1, C6—H6⋯O1 and C8—H8⋯O2 hydrogen bonds. Conformers *B* are linked along the *a-* and *b*-axis directions through C15—H15⋯O5, C18—H18⋯O5 and C12—H12⋯O7 inter­actions (Fig. 2 ▸, Table 1 ▸). The formed layers are joined by C5—H5⋯O7 and C19—H19⋯O2 hydrogen bonds.

## 4. Hirshfeld surface analysis

**Figure 3:**
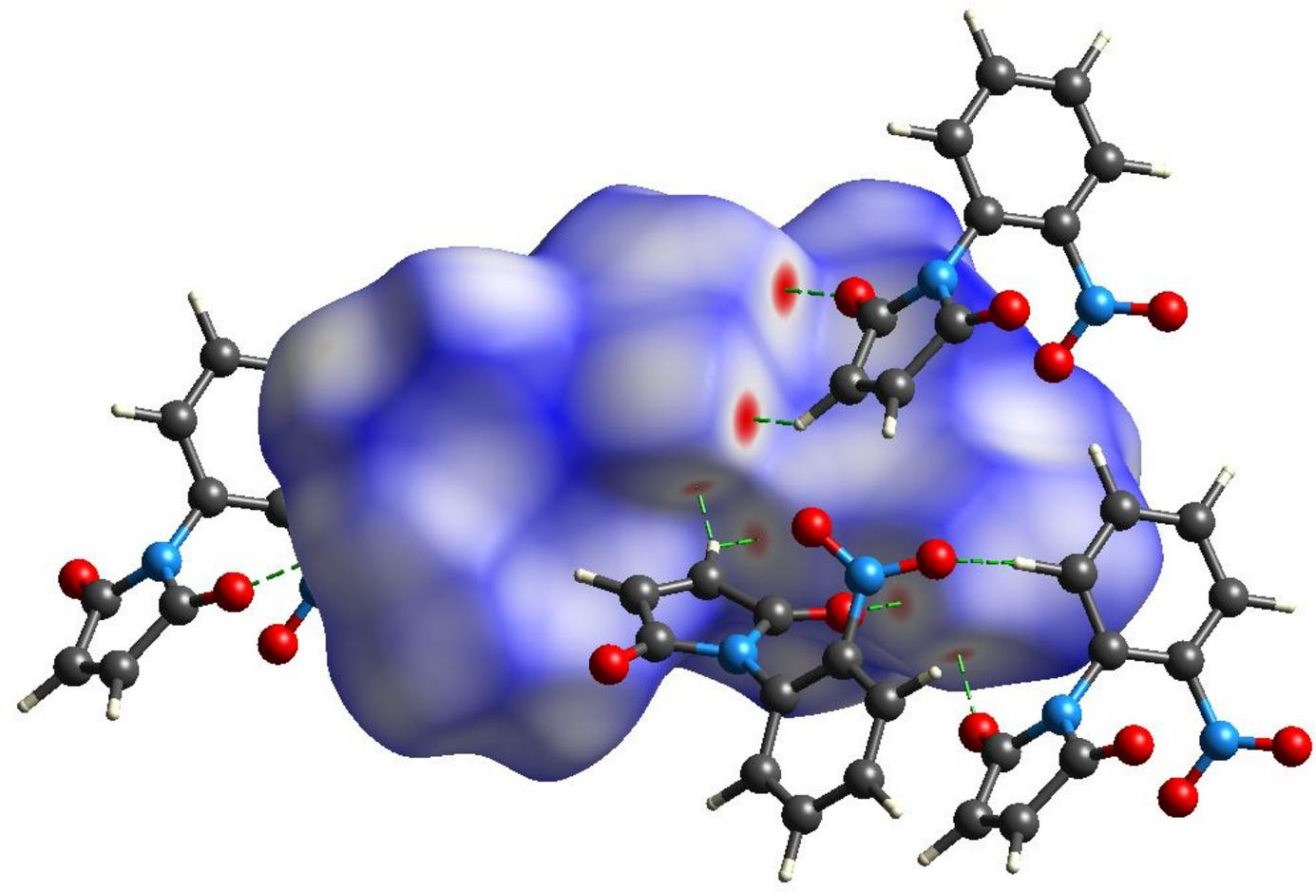
The Hirshfeld surface of the title compound mapped over *d*_norm_.

**Figure 4:**
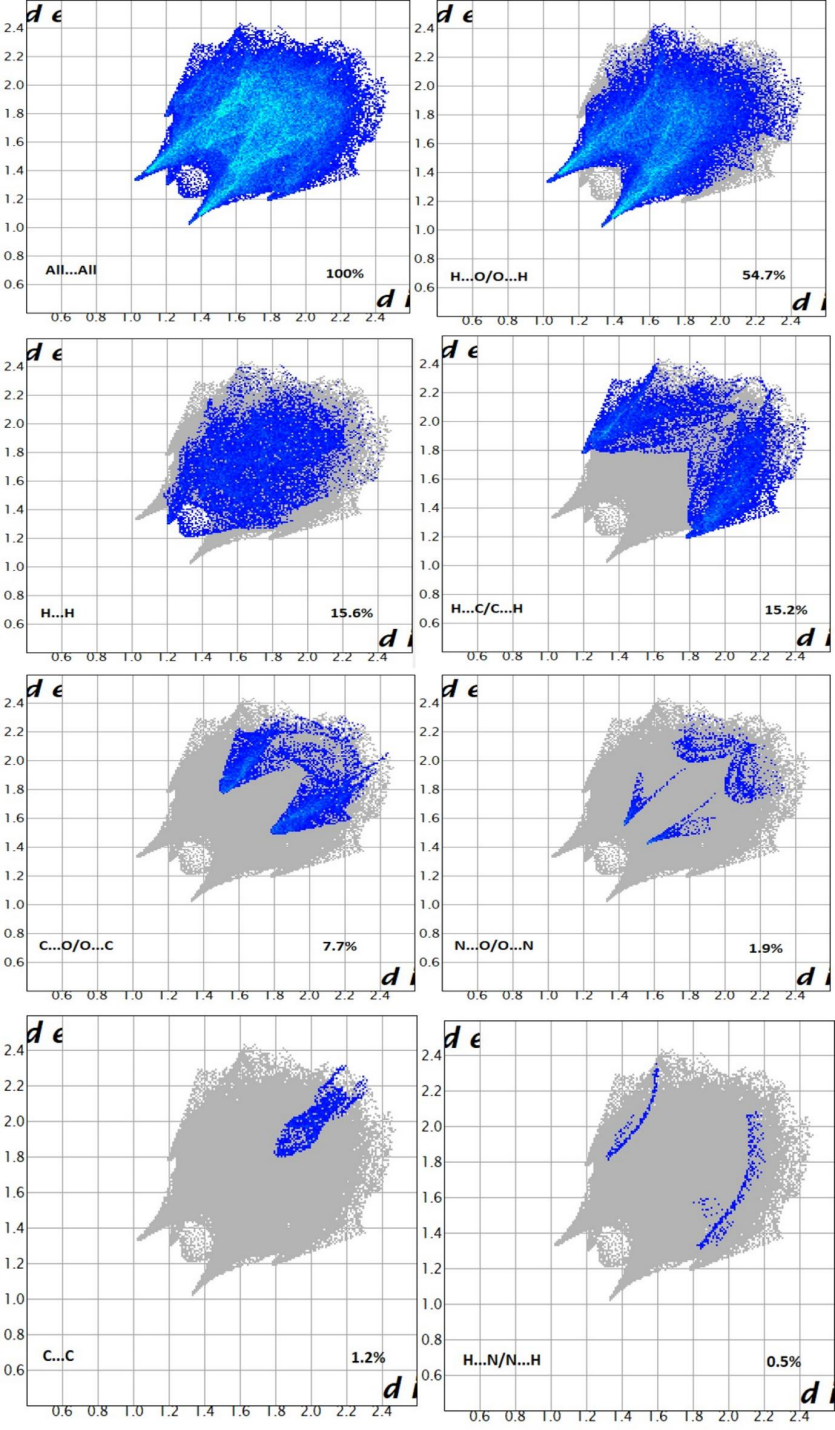
The fingerprint plots of the title compound delineated into the various labeled contacts.

A Hirshfeld surface analysis was performed using *CrystalExplorer 17.5 software* (Spackman *et al.*, 2021 ▸). Fig. 3 ▸ shows the Hirshfeld surface mapped over *d*_norm_ for the title compound, where red denotes shorter contacts (shorter than the sum of the van der Waals radii), blue denotes longer contacts (longer than the sum of the van der Waals radii), and white regions indicate contacts equal to the sum of the van der Waals radii. The red region in Fig. 3 ▸ represents the strongest and most important contacts represented by inter­molecular C—H⋯O hydrogen bonds. To qu­antify the supra­molecular inter­actions that give rise to crystal packing, two-dimensional fingerprint plots (FPP) were generated and these are shown in in Fig. 4 ▸. The FPP analysis reveals that H⋯O/O⋯H (54.7%) H⋯C/C⋯H (15.2%), and H⋯H (15.6%) are the most important inter­actions responsible for the largest contributions to the crystal packing of the title compound.

## 5. Computational details and DFT calculations

**Figure 5:**
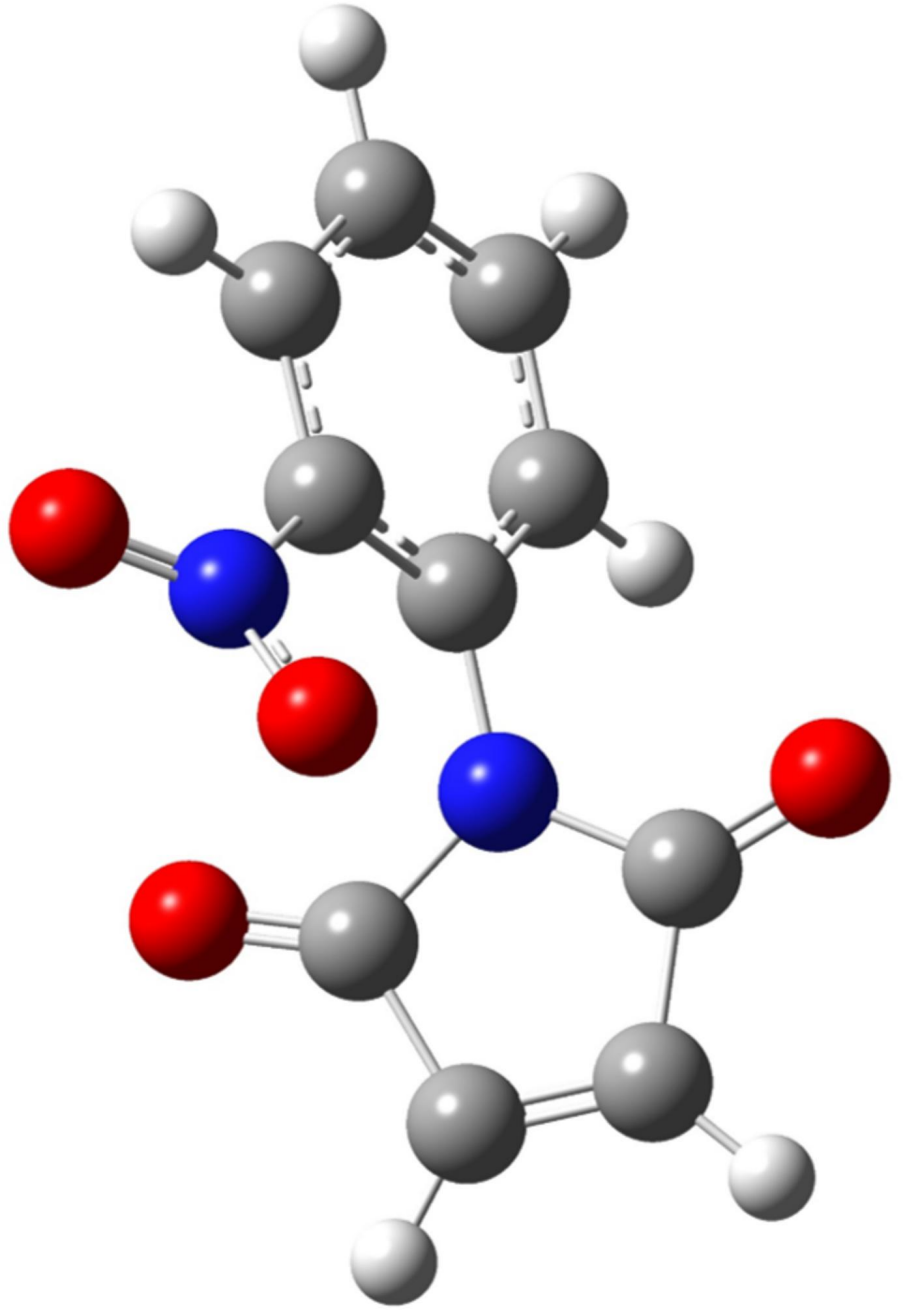
The optimized geometry of 1-(2-nitro­phen­yl)pyrrole-2,5-dione by (*a*) B3LYP/6–311+G (*d,p*); (*b*) wB97XD/Def2TZVPP and (*c*) LC-wpbe/6–311 g(2*d*,2*p*).

Computational quantum chemistry calculations were performed for 1-(2-nitro­phen­yl)pyrrole-2,5-dione using density functional theory (DFT). The following levels of theory were used to compare the change in stability, structural and electronic properties: B3LYP/6-311+G(d,p) (Clark *et al.*, 1983 ▸; Lee & Yang, 1988 ▸; Becke, 1993 ▸), *w*B97XD/Def2TZVPP (Weigend, & Ahlrichs, 2005 ▸; Chai & Head-Gordon, 2008 ▸), and LC-*w*pbe/6-311g(2d,2p) (Clark *et al.*, 1983 ▸; Vydrov *et al.*, 2006 ▸). For all calculations, the quantum chemistry *Gaussian 16* (Frisch *et al.*, 2019 ▸) software was employed. The mol­ecular geometries were fully optimized with a threshold of 10^−5^ a.u. for RMS forces. The optimized structures were confirmed to be true local minima by estimating the normal vibrations. Additionally, for 1-(2-nitro­phen­yl)pyrrole-2,5-dione, the potential energy curve was inspected through variations of the dihedral angle (C, N imide ring and C, C phenyl ring) at inter­vals of 15°, with the reference energy being the angle of 0° between the male­imide and benzene ring. At each point, the three levels of theory were used. The optimized geometry of 1-(2-nitro­phen­yl)pyrrole-2,5-dione is shown in Fig. 5 ▸. Some of the structural parameters such as bond lengths, bond angles, and the dihedral angle between the imide and phenyl rings are summarized in Table 2 ▸.

The structures present MPEs (Mean Percentage Errors) lower than 2.5% for the mean of the parameters compared to the three levels of theory evaluated. However, some values show a higher difference. For example, the C=C bond length of the male­imide ring is 0.024–0.011 Å longer compared to the experimental value. This discrepancy can be attributed to the presence of hydrogen-bonding inter­actions in the crystal structure of these mol­ecules. The dihedral angle is a crucial parameter that affects the properties of *N*-phenyl­male­imide derivatives (Cortes & Castro, 2016 ▸). For this structural parameter, a small difference was observed between the experimental angle and those obtained by DFT calculations. From the results obtained, it is concluded that the calculated structural parameters (lengths, bond angles and dihedral angle) using different levels of DFT theory agree excellently with the experimental data. Moreover, functionals including dispersion (wB97XD) and long-range correction (LC-wpbe) show values closer to those obtained experimentally.

**Figure 6:**
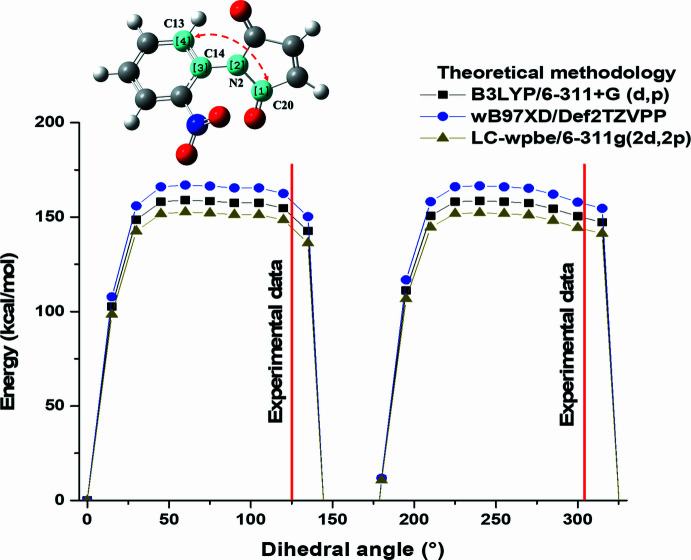
PES for 1-(2-nitro­phen­yl)pyrrole-2,5-dione with three DFT methods.

In 1-(2-nitro­phen­yl)pyrrole-2,5-dione, a repulsion is observed between the nitro group (NO_2_) and the oxygen atoms of the carbonyl group (C=O) of the male­imide ring, leading to a high value of the dihedral angle. To investigate this, the dihedral angle (C20—N2—C14—C15) of *N*-2-nitro­phenyl­male­imide was varied and the potential energy surface (PES) was determined using the three levels of theory. Fig. 6 ▸ illustrates a similar trend in the electronic energy values and zero-point energy corrections (ZPE) for all functionals used. For 1-(2-nitro­phen­yl)pyrrole-2,5-dione, a rotational barrier of approximately 150 kcal mol^−1^ determined with all three functionals was observed. This indicates the presence of a repulsive effect between NO_2_ and CO, resulting in rotamers with increased stability at specific spatial orientations. Inter­estingly, the angles observed in the crystalline dimers align with the most energetically stable rotamers calculated. Furthermore, the levels of theory incorporating including dispersion and long-range correction exhibit lower energetic values for each rotamer calculated (Fig. 5 ▸). These results are in agreement with previous studies using DFT methods (Cortes & Castro, 2016 ▸; Mao *et al.*, 2011 ▸).

## 6. Synthesis and crystallization

**Figure 7:**
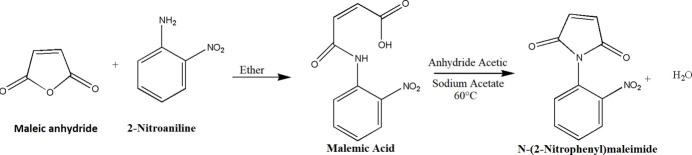
Reaction scheme for the synthesis of the title compound.

The synthesis of *o*-nitro­phenyl­maleimide was performed following the procedure described by Cava *et al.* (1961 ▸), which involves two steps (Fig. 7 ▸). In the first step, 2-nitro­(*N*-phen­yl)maleanilic acid was obtained by mixing 1.30 g of maleic anhydride and 25 mL of ethyl ether as solvent. Once the maleic anhydride was dissolved, a solution of *o*-nitro­aniline (1.83 g) in 5 mL of ether was added dropwise through the burette under constant stirring. The reaction mixture was stirred at room temperature for 1 h and then cooled in an ice bath. The product was obtained by vacuum filtration and used for the subsequent step of the synthesis. The reaction yield in the first step was 92%.

In the second step, *N*-(2-nitro­phen­yl)male­imide was obtained. In an Erlenmeyer flask, 16 mL of acetic anhydride and 1.30 g of sodium acetate anhydride were mixed. The previously obtained maleanilic acid (2.88 g) was then added to the reaction mixture under constant stirring and heating for 30 min. The reaction mixture was cooled to room temperature. The resulting solid was removed by vacuum filtration, washed three times with 5 mL portions of cool water and 5 mL of petroleum ether. 2.02 g of the compound were obtained in a yield in the second step of 76%. The final percentage yield in the synthesis was 69.85%.

Recrystallization was carried out using chloro­form, resulting in the formation of yellow prismatic crystals.

## 7. Refinement

Crystal data, data collection and structure refinement details are summarized in Table 3 ▸. Hydrogen atoms were positioned geometrically and refined using a riding model [C—H = 0.93 Å, *U*_iso_(H) = 1.2*U*_eq_(C)]. The positional disorder observed in the nitro group (O4*A*, O4*B*) was modeled by setting the occupancy factor to 0.5 for each atom.

## Supplementary Material

Crystal structure: contains datablock(s) I. DOI: 10.1107/S2056989024000926/vu2003sup1.cif

Structure factors: contains datablock(s) I. DOI: 10.1107/S2056989024000926/vu2003Isup2.hkl

Supporting information file. DOI: 10.1107/S2056989024000926/vu2003Isup3.cml

CCDC reference: 2328386

Additional supporting information:  crystallographic information; 3D view; checkCIF report

## Figures and Tables

**Table 1:** Hydrogen-bond geometry (Å, °)

*D*—H⋯*A*	*D*—H	H⋯*A*	*D*⋯*A*	*D*—H⋯*A*
C5—H5⋯O7^i^	0.93	2.64	3.457 (3)	148
C18—H18⋯O5^ii^	0.93	2.61	3.343 (3)	136
C16—H16⋯O3^iii^	0.93	2.79	3.210 (3)	109
C15—H15⋯O5^iv^	0.93	2.68	3.455 (3)	142
C19—H19⋯O2^v^	0.93	2.61	3.362 (3)	139
C19—H19⋯O6^vi^	0.93	2.61	3.388 (3)	141
C2—H2⋯O1^vii^	0.93	2.66	3.358 (3)	133

Symmetry codes: (i) 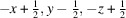 ; (ii) 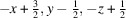 ; (iii) 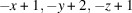 ; (iv) 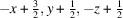 ; (v) 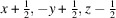 ; (vi) 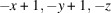 ; (vii) 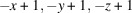 .

**Table 2:** Experimental and calculated bond lengths and angles (Å, °) of 1-(2-nitro­phen­yl)pyrrole-2,5-dione The numbering scheme used is that shown in Fig. 6 ▸.

Structural parameter	Calculation Method			Experimental
	B3LYP/6–311+G (d,p)	*w*B97XD/Def2TZVPP	LC-*w*pbe/6–311g(2d,2p)	
Bond lengths				
C8—C9, C18—C19	1.332	1.324	1.319	1.304, 1.308
C4—N1, C14—N2	1.416	1.409	1.409	1.416, 1.420
C7—O2, C10—O1, C20—O6, C17—O5	1.204, 1.205	1.197	1.198, 1200	1.200, 1.207
C3—N3, C13—N4	1.480	1.472	1.465	1.460, 1.465
N3—O3, N3—O4, N4—O7, N4—O8	1.221,1.225	1.209, 1.213	1.209, 1.212	1.261, 1.230, 1.222, 1.215
N1—C7, N1—C10, N2—C20, N2—C17	1.411, 1.412	1.399, 1.400	1.394, 1.395	1.388, 1.389, 1.392, 1.394
Mean percentage error (MPE)	0.949	0.516	0.283	
Bond angles				
C4—N1—C7, C4—N1—C10, C14—N2—C20, C14—N2—C17	124.7, 124.9	124.5, 124.7	124.9, 125.0	123.9, 125.6, 124.8, 125.2
O1—C10—C9, O2—C7—C8, O6—C20—C19, O5—C17—C18	128.6, 128.3	128.3, 128.5	128.5, 128.8	129.3, 129.5, 129.1, 129.2
C10—N1—C7, C20—N2—C17	110.2	110.4	110.1	109.6, 109.9
O3—N3—O4, O7—N4—O8	124.9	125.1	124.6	121.4.0, 124.0
N1—C4—C3, N2—C14—C13	123.0	122.7	122.5	122.7, 122.5
N1—C4—C5, N2—C14—C15	118.9	118.9	119.2	119.5, 119.2
Mean percentage error (MPE)	0.354	0.384	0.163	
Torsion angles				
C7—N1—C4—C3, C20—N2—C14—C15	123.2	124.6	125.6	100.0, 126.2
Mean percentage error (MPE)	2.377	1.268	0.475	

**Table 3:** Experimental details

Crystal data	
Chemical formula	C_10_H_6_N_2_O_4_
*M*_r_	218.17
Crystal system, space group	Monoclinic, *P*2_1_/*n*
Temperature (K)	293
*a*, *b*, *c* (Å)	14.331 (5), 7.769 (5), 17.558 (5)
β (°)	91.969 (5)
*V* (Å^3^)	1953.7 (15)
*Z*	8
Radiation type	Mo *K*α
μ (mm^−1^)	0.12
Crystal size (mm)	0.60 × 0.51 × 0.36

Data collection	
Diffractometer	Xcalibur, Atlas, Gemini
Absorption correction	Analytical (*CrysAlis PRO*; Agilent, 2012 ▸)
*T*_min_, *T*_max_	0.983, 0.988
No. of measured, independent and observed [*I* > 2σ(*I*)] reflections	8808, 5183, 2815
*R*_int_	0.020
(sin θ/λ)_max_ (Å^−1^)	0.681

Refinement	
*R*[*F* ^2^ > 2σ(*F* ^2^)], *wR*(*F* ^2^), *S*	0.048, 0.122, 1.02
No. of reflections	4548
No. of parameters	299
H-atom treatment	H-atom parameters constrained
Δρ_max_, Δρ_min_ (e Å^−3^)	0.21, −0.19

Computer programs: *CrysAlis PRO* (Agilent, 2012 ▸), *SHELXT2014/5* (Sheldrick, 2015*a* ▸), *SHELXL2016/6* (Sheldrick, 2015*b* ▸) and *OLEX2* (Dolomanov *et al.*, 2009 ▸).

## supplementary crystallographic information

### Crystal data

**Table:**

C_10_H_6_N_2_O_4_	*F*(000) = 896
*M**_r_* = 218.17	*D*_x_ = 1.483 Mg m^−^^3^
Monoclinic, *P*2_1_/*n*	Mo *K*α radiation, λ = 0.71073 Å
*a* = 14.331 (5) Å	Cell parameters from 2214 reflections
*b* = 7.769 (5) Å	θ = 3.6–29.5°
*c* = 17.558 (5) Å	µ = 0.12 mm^−^^1^
β = 91.969 (5)°	*T* = 293 K
*V* = 1953.7 (15) Å^3^	Needle, clear yellow
*Z* = 8	0.60 × 0.51 × 0.36 mm

### Data collection

**Table:**

Xcalibur, Atlas, Gemini diffractometer	*R*_int_ = 0.020
Radiation source: Enhance (Mo) X-ray Source	θ_max_ = 29.0°, θ_min_ = 3.6°
ω scans	*h* = −18→17
Absorption correction: analytical (CrysAlisPro; Agilent, 2012)	*k* = −7→10
*T*_min_ = 0.983, *T*_max_ = 0.988	*l* = −16→23
8808 measured reflections	3 standard reflections every 60 min
5183 independent reflections	intensity decay: none
2815 reflections with *I* > 2σ(*I*)	

### Refinement

**Table:**

Refinement on *F*^2^	Hydrogen site location: inferred from neighbouring sites
Least-squares matrix: full	H-atom parameters constrained
*R*[*F*^2^ > 2σ(*F*^2^)] = 0.048	*w* = 1/[σ^2^(*F*_o_^2^) + (0.0388*P*)^2^ + 0.6076*P*] where *P* = (*F*_o_^2^ + 2*F*_c_^2^)/3
*wR*(*F*^2^) = 0.122	(Δ/σ)_max_ < 0.001
*S* = 1.02	Δρ_max_ = 0.21 e Å^−^^3^
4548 reflections	Δρ_min_ = −0.19 e Å^−^^3^
299 parameters	Extinction correction: *SHELXL2016/6* (Sheldrick, 2015b), Fc^*^=kFc[1+0.001xFc^2^λ^3^/sin(2θ)]^-1/4^
0 restraints	Extinction coefficient: 0.0083 (8)
Primary atom site location: dual	

### Special details

**Table:**

Geometry. All esds (except the esd in the dihedral angle between two l.s. planes) are estimated using the full covariance matrix. The cell esds are taken into account individually in the estimation of esds in distances, angles and torsion angles; correlations between esds in cell parameters are only used when they are defined by crystal symmetry. An approximate (isotropic) treatment of cell esds is used for estimating esds involving l.s. planes.

### Fractional atomic coordinates and isotropic or equivalent isotropic displacement parameters (Å^2^)

**Table:**

	*x*	*y*	*z*	*U*_iso_*/*U*_eq_	Occ. (<1)
N2	0.57741 (10)	0.7048 (2)	0.17503 (8)	0.0448 (4)	
O6	0.47003 (10)	0.6430 (2)	0.07816 (8)	0.0720 (5)	
O8	0.42833 (12)	0.53297 (19)	0.23870 (10)	0.0726 (5)	
N1	0.23720 (10)	0.4967 (2)	0.46284 (9)	0.0476 (4)	
O5	0.70442 (11)	0.6767 (2)	0.25807 (9)	0.0809 (5)	
N4	0.38702 (12)	0.6666 (2)	0.22501 (9)	0.0521 (4)	
O2	0.11155 (11)	0.4289 (2)	0.53495 (10)	0.0818 (5)	
O7	0.30425 (11)	0.6748 (2)	0.20630 (10)	0.0807 (5)	
O1	0.33431 (11)	0.6568 (3)	0.38930 (11)	0.0936 (6)	
C13	0.43816 (12)	0.8286 (2)	0.23277 (10)	0.0389 (4)	
C14	0.53010 (12)	0.8421 (2)	0.21045 (10)	0.0396 (4)	
N3	0.37727 (17)	0.6062 (3)	0.57413 (12)	0.0727 (6)	
O4A	0.3015 (11)	0.6806 (17)	0.5880 (10)	0.129 (5)	0.5
C4	0.30211 (13)	0.3785 (3)	0.49581 (10)	0.0458 (5)	
C12	0.39261 (14)	0.9659 (2)	0.26434 (11)	0.0493 (5)	
H12	0.330597	0.955447	0.277613	0.059*	
C15	0.57553 (14)	0.9976 (3)	0.22127 (11)	0.0505 (5)	
H15	0.636591	1.010733	0.205935	0.061*	
C20	0.54333 (14)	0.6143 (3)	0.11138 (11)	0.0514 (5)	
C17	0.66103 (13)	0.6319 (3)	0.20183 (12)	0.0531 (5)	
C3	0.37054 (14)	0.4268 (3)	0.54888 (11)	0.0523 (5)	
C7	0.14624 (14)	0.5155 (3)	0.48641 (12)	0.0538 (5)	
C11	0.43925 (16)	1.1178 (3)	0.27605 (12)	0.0573 (5)	
H11	0.409483	1.210544	0.298310	0.069*	
C16	0.53049 (16)	1.1328 (3)	0.25470 (12)	0.0598 (6)	
H16	0.562129	1.236008	0.263028	0.072*	
C10	0.25829 (15)	0.6291 (3)	0.41321 (12)	0.0579 (6)	
O3	0.45594 (16)	0.6661 (3)	0.58559 (13)	0.1184 (8)	
C19	0.61368 (15)	0.4812 (3)	0.09608 (13)	0.0609 (6)	
H19	0.610624	0.403119	0.055928	0.073*	
C9	0.17067 (16)	0.7259 (3)	0.40028 (13)	0.0652 (6)	
H9	0.162690	0.819245	0.367519	0.078*	
C5	0.29837 (16)	0.2077 (3)	0.47417 (13)	0.0640 (6)	
H5	0.253871	0.171906	0.437746	0.077*	
C18	0.68104 (16)	0.4907 (3)	0.14809 (13)	0.0639 (6)	
H18	0.733352	0.419827	0.150902	0.077*	
C8	0.10613 (15)	0.6601 (3)	0.44202 (13)	0.0652 (6)	
H8	0.044719	0.698340	0.443510	0.078*	
C2	0.43382 (16)	0.3107 (4)	0.57970 (13)	0.0720 (7)	
H2	0.480623	0.346376	0.614146	0.086*	
C1	0.42634 (19)	0.1411 (4)	0.55849 (16)	0.0805 (8)	
H1	0.467045	0.060191	0.580155	0.097*	
C6	0.3598 (2)	0.0902 (3)	0.50596 (16)	0.0802 (8)	
H6	0.355932	−0.024794	0.491543	0.096*	
O4B	0.3109 (8)	0.6914 (10)	0.5784 (7)	0.076 (2)	0.5

### Atomic displacement parameters (Å^2^)

**Table:**

	*U*^11^	*U*^22^	*U*^33^	*U*^12^	*U*^13^	*U*^23^
N2	0.0371 (8)	0.0495 (9)	0.0474 (9)	0.0031 (7)	−0.0032 (6)	−0.0111 (8)
O6	0.0586 (9)	0.0980 (13)	0.0582 (9)	0.0062 (9)	−0.0134 (7)	−0.0229 (9)
O8	0.0853 (12)	0.0376 (8)	0.0959 (12)	−0.0044 (8)	0.0160 (9)	0.0038 (8)
N1	0.0399 (9)	0.0537 (10)	0.0494 (9)	0.0047 (7)	0.0030 (7)	0.0106 (8)
O5	0.0592 (9)	0.1078 (14)	0.0739 (10)	0.0278 (9)	−0.0224 (8)	−0.0266 (10)
N4	0.0487 (10)	0.0508 (11)	0.0569 (10)	−0.0118 (8)	0.0034 (8)	0.0013 (8)
O2	0.0567 (10)	0.0974 (13)	0.0925 (12)	−0.0001 (9)	0.0200 (8)	0.0336 (11)
O7	0.0474 (9)	0.0968 (13)	0.0973 (12)	−0.0276 (9)	−0.0077 (8)	−0.0006 (10)
O1	0.0575 (10)	0.1259 (16)	0.0987 (13)	0.0074 (10)	0.0215 (9)	0.0615 (12)
C13	0.0386 (9)	0.0354 (9)	0.0425 (9)	−0.0017 (8)	−0.0025 (7)	0.0041 (8)
C14	0.0363 (9)	0.0416 (10)	0.0405 (9)	−0.0002 (8)	−0.0027 (7)	−0.0025 (8)
N3	0.0769 (15)	0.0820 (16)	0.0584 (12)	−0.0005 (14)	−0.0112 (11)	−0.0154 (11)
O4A	0.135 (9)	0.133 (9)	0.119 (7)	0.036 (6)	0.025 (5)	−0.042 (6)
C4	0.0436 (10)	0.0491 (11)	0.0451 (10)	0.0054 (9)	0.0065 (8)	0.0069 (9)
C12	0.0442 (11)	0.0474 (11)	0.0566 (12)	0.0075 (9)	0.0058 (9)	0.0066 (10)
C15	0.0451 (11)	0.0543 (12)	0.0522 (11)	−0.0121 (10)	0.0026 (8)	−0.0039 (10)
C20	0.0498 (11)	0.0568 (12)	0.0476 (11)	−0.0055 (10)	0.0018 (9)	−0.0089 (10)
C17	0.0433 (11)	0.0624 (13)	0.0533 (12)	0.0083 (10)	−0.0013 (9)	−0.0057 (10)
C3	0.0533 (12)	0.0610 (13)	0.0428 (10)	0.0100 (10)	0.0022 (9)	0.0003 (10)
C7	0.0421 (11)	0.0620 (13)	0.0575 (12)	−0.0005 (10)	0.0046 (9)	0.0073 (11)
C11	0.0709 (14)	0.0407 (11)	0.0609 (13)	0.0093 (11)	0.0090 (11)	−0.0009 (10)
C16	0.0753 (15)	0.0423 (11)	0.0618 (13)	−0.0159 (11)	0.0035 (11)	−0.0075 (10)
C10	0.0515 (12)	0.0702 (14)	0.0520 (12)	0.0017 (11)	0.0024 (9)	0.0176 (11)
O3	0.1028 (16)	0.1317 (19)	0.1193 (17)	−0.0274 (14)	−0.0180 (13)	−0.0397 (14)
C19	0.0650 (14)	0.0576 (13)	0.0609 (13)	−0.0005 (11)	0.0105 (11)	−0.0191 (11)
C9	0.0606 (14)	0.0675 (15)	0.0669 (14)	0.0084 (12)	−0.0075 (11)	0.0223 (12)
C5	0.0690 (15)	0.0547 (13)	0.0681 (14)	−0.0004 (11)	0.0015 (11)	0.0029 (12)
C18	0.0601 (14)	0.0630 (14)	0.0690 (14)	0.0163 (11)	0.0096 (11)	−0.0083 (12)
C8	0.0474 (12)	0.0747 (15)	0.0735 (14)	0.0156 (12)	0.0007 (11)	0.0109 (13)
C2	0.0615 (14)	0.100 (2)	0.0540 (13)	0.0235 (14)	−0.0013 (10)	0.0112 (14)
C1	0.0797 (18)	0.086 (2)	0.0766 (17)	0.0393 (16)	0.0147 (14)	0.0276 (16)
C6	0.095 (2)	0.0514 (14)	0.0954 (19)	0.0169 (14)	0.0168 (16)	0.0094 (14)
O4B	0.081 (5)	0.040 (3)	0.106 (6)	0.015 (3)	−0.021 (4)	−0.014 (3)

### Geometric parameters (Å, º)

**Table:**

N2—C14	1.419 (2)	C15—H15	0.9300
N2—C20	1.394 (2)	C15—C16	1.375 (3)
N2—C17	1.393 (2)	C20—C19	1.475 (3)
O6—C20	1.205 (2)	C17—C18	1.481 (3)
O8—N4	1.215 (2)	C3—C2	1.377 (3)
N1—C4	1.416 (2)	C7—C8	1.473 (3)
N1—C7	1.389 (2)	C11—H11	0.9300
N1—C10	1.388 (3)	C11—C16	1.377 (3)
O5—C17	1.200 (2)	C16—H16	0.9300
N4—O7	1.222 (2)	C10—C9	1.475 (3)
N4—C13	1.460 (2)	C19—H19	0.9300
O2—C7	1.207 (2)	C19—C18	1.308 (3)
O1—C10	1.200 (2)	C9—H9	0.9300
C13—C14	1.391 (2)	C9—C8	1.304 (3)
C13—C12	1.377 (3)	C5—H5	0.9300
C14—C15	1.382 (3)	C5—C6	1.373 (3)
N3—O4A	1.261 (15)	C18—H18	0.9300
N3—C3	1.465 (3)	C8—H8	0.9300
N3—O3	1.230 (3)	C2—H2	0.9300
N3—O4B	1.164 (11)	C2—C1	1.372 (4)
C4—C3	1.381 (3)	C1—H1	0.9300
C4—C5	1.381 (3)	C1—C6	1.362 (4)
C12—H12	0.9300	C6—H6	0.9300
C12—C11	1.369 (3)		
			
C20—N2—C14	124.81 (15)	C2—C3—C4	122.0 (2)
C17—N2—C14	125.19 (15)	N1—C7—C8	106.01 (18)
C17—N2—C20	109.84 (16)	O2—C7—N1	124.43 (19)
C7—N1—C4	123.81 (16)	O2—C7—C8	129.5 (2)
C10—N1—C4	125.65 (16)	C12—C11—H11	120.2
C10—N1—C7	109.61 (16)	C12—C11—C16	119.69 (19)
O8—N4—O7	124.03 (18)	C16—C11—H11	120.2
O8—N4—C13	118.58 (16)	C15—C16—C11	121.02 (19)
O7—N4—C13	117.37 (18)	C15—C16—H16	119.5
C14—C13—N4	121.04 (16)	C11—C16—H16	119.5
C12—C13—N4	117.50 (16)	N1—C10—C9	105.79 (18)
C12—C13—C14	121.45 (17)	O1—C10—N1	124.88 (19)
C13—C14—N2	122.56 (16)	O1—C10—C9	129.3 (2)
C15—C14—N2	119.17 (16)	C20—C19—H19	125.5
C15—C14—C13	118.24 (17)	C18—C19—C20	109.02 (19)
O4A—N3—C3	116.5 (7)	C18—C19—H19	125.5
O3—N3—O4A	125.8 (7)	C10—C9—H9	125.4
O3—N3—C3	117.4 (2)	C8—C9—C10	109.3 (2)
O4B—N3—C3	121.0 (4)	C8—C9—H9	125.4
O4B—N3—O3	121.4 (5)	C4—C5—H5	119.7
C3—C4—N1	122.75 (18)	C6—C5—C4	120.6 (2)
C5—C4—N1	119.45 (18)	C6—C5—H5	119.7
C5—C4—C3	117.80 (19)	C17—C18—H18	125.3
C13—C12—H12	120.2	C19—C18—C17	109.39 (19)
C11—C12—C13	119.52 (18)	C19—C18—H18	125.3
C11—C12—H12	120.2	C7—C8—H8	125.5
C14—C15—H15	120.0	C9—C8—C7	108.97 (19)
C16—C15—C14	120.04 (19)	C9—C8—H8	125.5
C16—C15—H15	120.0	C3—C2—H2	120.7
N2—C20—C19	106.06 (17)	C1—C2—C3	118.6 (2)
O6—C20—N2	124.78 (19)	C1—C2—H2	120.7
O6—C20—C19	129.16 (19)	C2—C1—H1	119.7
N2—C17—C18	105.62 (17)	C6—C1—C2	120.6 (2)
O5—C17—N2	125.12 (19)	C6—C1—H1	119.7
O5—C17—C18	129.25 (19)	C5—C6—H6	119.8
C4—C3—N3	120.01 (19)	C1—C6—C5	120.4 (2)
C2—C3—N3	118.0 (2)	C1—C6—H6	119.8
			
N2—C14—C15—C16	179.26 (18)	C4—N1—C10—C9	−174.82 (18)
N2—C20—C19—C18	1.9 (3)	C4—C3—C2—C1	−2.1 (3)
N2—C17—C18—C19	−1.3 (3)	C4—C5—C6—C1	−1.1 (4)
O6—C20—C19—C18	−177.2 (2)	C12—C13—C14—N2	−177.44 (17)
O8—N4—C13—C14	38.8 (2)	C12—C13—C14—C15	0.5 (3)
O8—N4—C13—C12	−139.90 (19)	C12—C11—C16—C15	0.4 (3)
N1—C4—C3—N3	0.9 (3)	C20—N2—C14—C13	51.8 (3)
N1—C4—C3—C2	−179.20 (19)	C20—N2—C14—C15	−126.1 (2)
N1—C4—C5—C6	−179.2 (2)	C20—N2—C17—O5	−176.2 (2)
N1—C7—C8—C9	−3.9 (3)	C20—N2—C17—C18	2.5 (2)
N1—C10—C9—C8	3.0 (3)	C20—C19—C18—C17	−0.4 (3)
O5—C17—C18—C19	177.4 (3)	C17—N2—C14—C13	−123.1 (2)
N4—C13—C14—N2	3.9 (3)	C17—N2—C14—C15	59.0 (3)
N4—C13—C14—C15	−178.18 (16)	C17—N2—C20—O6	176.4 (2)
N4—C13—C12—C11	176.92 (17)	C17—N2—C20—C19	−2.8 (2)
O2—C7—C8—C9	174.7 (2)	C3—C4—C5—C6	1.3 (3)
O7—N4—C13—C14	−142.47 (18)	C3—C2—C1—C6	2.4 (4)
O7—N4—C13—C12	38.8 (2)	C7—N1—C4—C3	−100.0 (2)
O1—C10—C9—C8	−174.7 (3)	C7—N1—C4—C5	80.6 (3)
C13—C14—C15—C16	1.3 (3)	C7—N1—C10—O1	172.4 (2)
C13—C12—C11—C16	1.3 (3)	C7—N1—C10—C9	−5.5 (2)
C14—N2—C20—O6	0.9 (3)	C10—N1—C4—C3	67.8 (3)
C14—N2—C20—C19	−178.29 (17)	C10—N1—C4—C5	−111.6 (2)
C14—N2—C17—O5	−0.7 (3)	C10—N1—C7—O2	−172.8 (2)
C14—N2—C17—C18	178.04 (18)	C10—N1—C7—C8	5.9 (2)
C14—C13—C12—C11	−1.8 (3)	C10—C9—C8—C7	0.5 (3)
C14—C15—C16—C11	−1.7 (3)	O3—N3—C3—C4	−142.3 (2)
N3—C3—C2—C1	177.8 (2)	O3—N3—C3—C2	37.8 (3)
O4A—N3—C3—C4	43.2 (9)	C5—C4—C3—N3	−179.7 (2)
O4A—N3—C3—C2	−136.7 (9)	C5—C4—C3—C2	0.2 (3)
C4—N1—C7—O2	−3.3 (3)	C2—C1—C6—C5	−0.9 (4)
C4—N1—C7—C8	175.37 (18)	O4B—N3—C3—C4	32.9 (8)
C4—N1—C10—O1	3.1 (4)	O4B—N3—C3—C2	−147.0 (8)

### Hydrogen-bond geometry (Å, º)

**Table:**

*D*—H···*A*	*D*—H	H···*A*	*D*···*A*	*D*—H···*A*
C5—H5···O7^i^	0.93	2.64	3.457 (3)	148
C18—H18···O5^ii^	0.93	2.61	3.343 (3)	136
C16—H16···O3^iii^	0.93	2.79	3.210 (3)	109
C15—H15···O5^iv^	0.93	2.68	3.455 (3)	142
C19—H19···O2^v^	0.93	2.61	3.362 (3)	139
C19—H19···O6^vi^	0.93	2.61	3.388 (3)	141
C2—H2···O1^vii^	0.93	2.66	3.358 (3)	133

Symmetry codes: (i) −*x*+1/2, *y*−1/2, −*z*+1/2; (ii) −*x*+3/2, *y*−1/2, −*z*+1/2; (iii) −*x*+1, −*y*+2, −*z*+1; (iv) −*x*+3/2, *y*+1/2, −*z*+1/2; (v) *x*+1/2, −*y*+1/2, *z*−1/2; (vi) −*x*+1, −*y*+1, −*z*; (vii) −*x*+1, −*y*+1, −*z*+1.
